# Plant–herbivore interactions: Combined effect of groundwater level, root vole grazing, and sedge silicification

**DOI:** 10.1002/ece3.8275

**Published:** 2021-10-30

**Authors:** Zbigniew Borowski, Karol Zub, Marcin Sulwiński, Małgorzata Suska‐Malawska, Marek Konarzewski

**Affiliations:** ^1^ Department of Forest Ecology Forest Research Institute Raszyn Poland; ^2^ Mammal Research Institute Polish Academy of Sciences Białowieża Poland; ^3^ Faculty of Biology, Biological and Chemical Research Centre Warsaw University Warsaw Poland; ^4^ Faculty of Biology University in Białystok Białystok Poland

**Keywords:** groundwater level, plant defense, population density, tussock sedges, voles

## Abstract

1. Accumulation of silica (Si) by plants can be driven by (1) herbivory pressure (and therefore plant–herbivore interactions), (2) geohydrological cycles, or (3) a combination of (1) and (2), with (1–3) possibly affecting Si concentration with a 1‐year delay.
2. To identify the relative significance of (1–3), we analyzed the concentration of Si in fibrous tussock sedge (*Carex appropinquata*), the population density of the root vole (*Microtus oeconomus*), and the groundwater level, over 11 years.
3. The largest influence of autumn Si concentration in leaves (Si_leaf_) was on the level of the current‐year groundwater table, which was positive and accounted for 13.3% of its variance. The previous year's vole population density was weakly positively correlated with Si_leaf_, and it alone explained 9.5% of its variance.
4. The only variable found to have a positive, significant effect on autumn Si concentration in rhizomes (Si_rhiz_) was the current‐year spring water level, explaining as much as 60.9% of its variance.
5. We conclude that the changes in Si concentration in fibrous tussock sedge are predominantly driven by hydrology, with vole population dynamics being secondary.
6. Our results provide only partial support for the existence of plant–herbivore interactions, as we did not detect the significant effects of Si tussock concentration on the vole density dynamics. This was mainly due to the low level of silicification of sedges, which was insufficient to impinge herbivores.
7. Future studies on plant–herbivore interactions should therefore aim at disentangling whether anti‐herbivore protection is dependent on threshold values of herbivore population dynamics. Furthermore, studies on Si accumulation should focus on the effect of water‐mediated Si availability.

## Graphical abstract

To identify mechanisms of silica (Si) accumulation by plants, we analyzed Si concentration in fibrous tussock sedge (*Carex appropinquata*), the root vole (*Microtus oeconomus*) population density, and water table over 11 years. Changes in Si concentration in fibrous tussock sedge are predominantly driven by hydrology, whereas vole population dynamics are secondary. Our results only partially support the existence of plant–herbivore interactions, as we did not detect significant effects of Si tussock concentration on the vole population dynamics. This was mainly due to the low degree of silicification of sedges, which was insufficient to impinge herbivores.

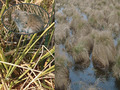

## 1 INTRODUCTION

Recent studies suggest that the dynamics of the small mammal population are shaped primarily by the external factors (the so‐called trophic interactions) such as predation and availability or quality of food (Klemola et al., 2000; Lambin et al., 2000; Oli, 2019, but see Andreassen et al., 2013). Among factors related to food quality, root and leaf silicification induced by past overgrazing has recently received particular attention. A number of studies demonstrated that abrasive properties of silicon contained in the plant tissues deteriorate herbivore teeth (Calandra et al., 2016), cause abrasion of intestinal villi (Wieczorek et al., 2015), and reduce body mass and survival prospects (Wieczorek et al., 2015; Zub et al., 2014). Furthermore, plant responses to herbivory pressure have been observed within laboratory‐based studies, in which pressure from field voles (*Microtus agrestis*) increased the presence of silicon in grass tissues by 400% (Massey & Hartley, 2006).

Yet, the results of the field studies on the associations between induced silicon plant defense and mammalian herbivory have not been as clear as the laboratory ones. Studies conducted in Norway have shown that the induced grass response to the herbivory manifested by an increase in the content of Si in plant tissues is extremely variable and depends not only on the pressure of herbivorous mammals (rodents and reindeer) but also on the location, plant species, and its genotype (Soininen et al., 2013). Quigley et al. (2020) demonstrated that Si concentration in grass leaves did not respond to large mammalian grazer exclusion studied in a climatic gradient, but it was strongly affected by nutrient availability. In turn, field experiments carried out in Kielder Forest (UK) showed that after several months of density manipulation, the level of silicon in wavy hair grass (*Deschampsia caespitosa*) leaves decreased by 22% on sites where field vole density had been reduced, but the increase in silicon content did not affect body weight of voles nor their spring population growth rate or survival, which suggests that plant quality hypothesis is unlikely to explain the observed cyclicity in the Kielder Forest field vole population (Ruffino et al., 2018). Likewise, Wieczorek, Zub, et al. (2015) (but see Soininen et al., 2017) showed that vole herbivory elevated silicon levels in sedges, albeit with no detectable effect on the winter survival rates of voles.

The above inconsistencies may simply stem from the lack of sufficient statistical power of these analyses, since the longest time‐series analyses lasted between 3 (Soininen et al., 2013) and 4 years (Wieczorek, Zub, et al., 2015) and are therefore based on a small number of degrees of freedom. Long‐term studies, able to capture the time course of the putative plant–herbivore association, are particularly needed because changes in silicon levels in plant tissues are not only due to grazing but are also responsive to abiotic factors, chiefly water availability, which drives silicon absorption in the form of silicic acid (Brightly et al., 2020; Faisal et al., 2012; Kindomihou et al., 2006; Raven, 1983; Sangster et al., 2001). The effect of the water on the induction of silicon in plants has been indirectly demonstrated in sedge leaves in European ecosystems (Wieczorek, Zub, et al., 2015) and in leaves of two African grass species (Quigley & Anderson, 2014).

To address such short‐term limitations in previous research, we analyzed a 11‐year time series of: groundwater level; population dynamics of the root vole (*Microtus oeconomus*); and silicon levels in the tissues of the fibrous tussock sedges (*Carex appropinquata*, Schumacher, 1801)—the main food source of the voles. To our knowledge, this is the longest time series ever used to test the effect of plant defenses and water availability in plant–herbivore interactions. We tested whether: sedges induce silicon defenses in response to the grazing by root voles; the feedback of silicon in sedges influences vole population dynamics; and the groundwater level influenced the plant–herbivore system. Following Wieczorek, Zub, et al. (2015), we did so by taking into consideration Si concentration in rhizomes (Si_rhiz_) and in leaves (Si_leaf_), as the dynamics of the plant–herbivore interaction can be different depending on the part of the plant. We predicted that Si_leaf_ should be positively affected by the previous year's vole population density. However, Si_rhiz_ should be primarily stimulated by the same year herbivore‐incurred damage. As Si uptake by sedges is positively driven by water availability, we also surmised that Si_leaf_ and Si_rhiz_ were likely to be positively affected by the level of groundwater table in previous and/or current spring. Conversely, year‐to‐year changes in vole population density should be inversely correlated with the Si_leaf_ and Si_rhiz_ with a one‐year time lag.

## 2 STUDY AREA

The study was conducted in the Lower Basin of the Biebrza National Park, NE Poland (53°36′18″N, 22°55′36″E). The study area is located in a homogenous sedge wetland with vegetation dominated by plants from *Cyperaceae* family. The main plant species in the Park is the fibrous tussock sedge, which covers 85% of the area and forms hummock–hollow structures (Matuszkiewicz, 2020). The wetland has a seasonal water regime with the highest level during spring, when flooding is frequent. The climate is characterized by long winters (>100 days), short and early springs, and short summers (77–85 days).

**FIGURE 1:**
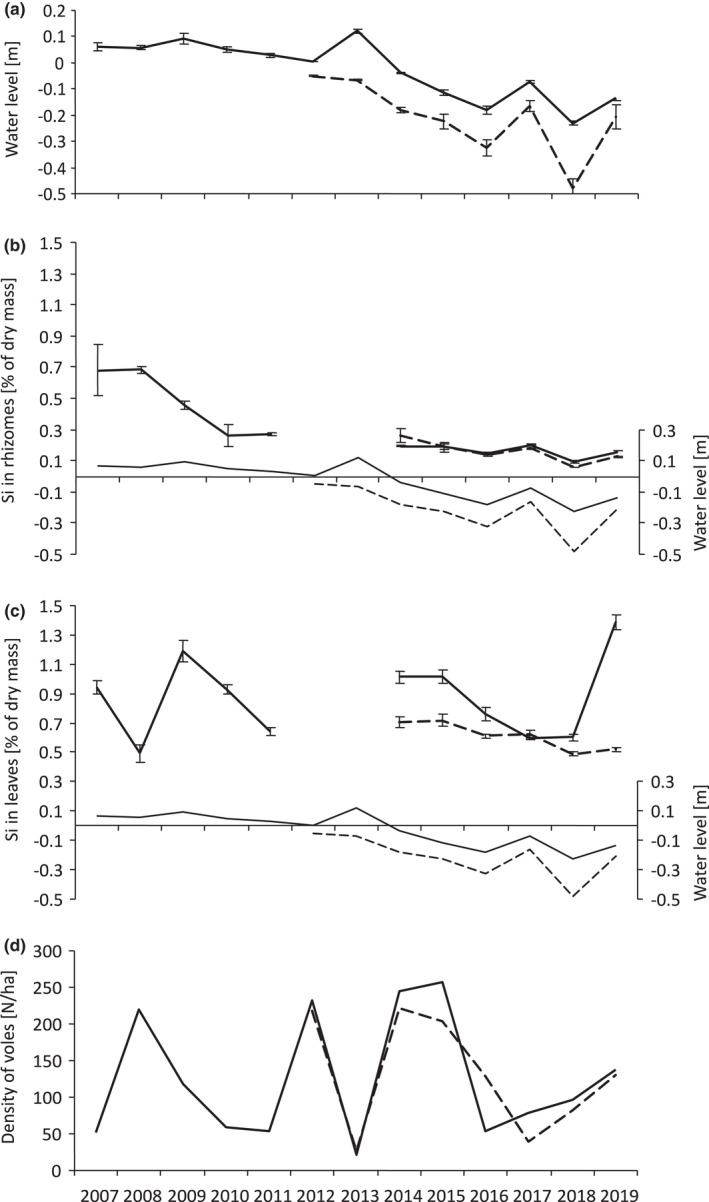
(a) Level of the groundwater (mean ± SE), concentration of Si (mean ± SE) in leaves (b) and rhizomes (c) of *Carex appropinquata*, and (d) autumn density of *Microtus oeconomus*, at the Gugny site (solid line) and the Barwik site (dashed line). For the sake of clarity and comprehension, on panels b and c, we added thin solid and dashed lines depicting water levels at the Gugny and Barwik sites, respectively

The main herbivores in the area are rodents and moose (*Alces alces*). Root voles are the dominant rodent species in this habitat, making up 90% of small mammal communities (Borowski, 2002, 2011). We worked with a natural population of root voles, which displays cyclical dynamics (Borowski, 2011). The study began in 2007, during which time various vole population peaks (2008–2009, 2015–2016) and crashes (2007, 2017) occurred (Figure 1).

### 2.1 Vole densities

In order to estimate vole population sizes, we carried out capture–mark–recapture (CMR) trapping free‐living populations in two sites, each 0.6 ha large and separated by 3 km. The first site, called Gugny, was trapped between 2007 and 2019, while the second, called Barwik, was trapped from 2012 to 2019. Trapping occurred at each site once a year in autumn (November). Throughout the study, we consistently monitored vole abundance with live traps. The trapping grids consisted of 77 traps spaced 10 m apart. We trapped at both study sites simultaneously. Traps were set for 5 days and checked twice a day. This schedule allowed us to avoid collateral trap mortality.

We marked each vole individually by toe clipping when it was first captured. Vole population size estimates were converted to density per ha based on the CMR method (see Borowski, 2011, for details).

### 2.2 Sedge sampling

Sedges are the main food of root voles, both in summer and in winter. In summer, the diet is dominated by green parts of the plant, whereas in late autumn and winter, voles also eat the woody parts, such as dry rhizomes and roots (Batzli & Henttonen, 1990; Gębczyńska, 1970; Tast, 1966).

In the Barwik and Gugny sites, every November from 2014 to 2019, we collected 10 haphazardly selected samples of sedge tussocks at each site. This resulted in 120 *Carex* tussocks samples (10 per year in each site over 6 years). To determine the Si concentration, from each tussock we took biomass sample composed of leaves or rhizomes produced in the present year, physically connected with the tussocks (representing a single plant). Dead parts of the plant or decaying litter was discarded. As voles do not feed on decaying litter, we selected only leaves and rhizomes that were both physically connected and composed of dried, non‐decomposing tissues. We separated leaves from rhizomes, and samples were cleaned under running water, dried at 80°C to a constant mass, and stored in separate plastic bags for further analysis.

Data on Si concentrations in leaves and rhizomes from the Gugny site (from 2007 to 2011) were collected in a similar manner as described above (for details, see Wieczorek, Zub, et al. (2015)). We calculated the mean value of Si concentration in leaves or rhizomes from 10 samples collected for each year and site; thus, our sample size was *N* = 17 (5 samples for period 2007–2011 from Gugny and 12 samples for period 2014–2019 from Gugny and Barwik).

### 2.3 Water level

We measured water level using piezometers, with the instruments in both study sites, each located ca. 5 km from the Biebrza River. Five to six measurements of water level (m) were taken in May and June using the same respective piezometer. These measurements were then averaged to be used later in analysis. The river and its floodplain form an interconnected spatially distributed system (Fisher et al., 1998) that experiences regular flooding, which occurs in spring.

### 2.4 Chemical analysis

The aboveground biomass and roots were separated and milled using a Tecator Cyclotec 1093 Mill. Each 150 mg sample of biomass was then digested in a 9:1 mixture of concentrated HNO_3_ and HF in Speedwave Four apparatus (Berghof), with temperatures reaching a maximum of 230°C. Si content in digested material was measured using an atomic absorption spectrometer, contrAA 700 (Analytik Jena), in nitrous oxide–acetylene flame with 251.6‐nm wavelength. Recovery of Si was determined using NCS DC73349 certified material (recovery was within 91% to 102% with a mean of 96%).

### 2.5 Statistical analyses

To analyze the data, we used generalized linear mixed models (GLMMs) with *log* link function. We log‐transformed all, but one (water level), variables to correct for their right‐skewed distribution. Model assumptions were checked using residual plots. These confirmed the following: ε was normally distributed, the model fits lacked heteroskedasticity, and no observations were disproportionately influential in any of the models.

To identify factors affecting the Si concentrations in leaves or rhizomes in November, we used the following variables, with their respective interactions, as the fixed terms: the autumn density of voles in the previous year (*t* − 1) and the current year (*t*), the spring water level from the present year (*t*), and Si concentration in leaves or rhizomes in the present year (*t*). In the final models, only the significant interaction terms were retained. The study site (Gugny or Barwik) and year of study were used as random factors. As the “study site” random factor has only two levels in some models, it caused singularity and then was removed. We used year as a random factor to resolve the problem of autocorrelation of Si concentration between year *t* and year *t* − 1.

We used similarly structured GLMM with *log* link function to analyze vole density in year n. The model included the following: Si concentration in the leaves and rhizomes (in year *t*); the previous year's vole density (year *t* − 1); the current (year *t*) water level as fixed effects. As with the *Si* models, site and year were included as a varying intercept random effect.

For all models, we calculated the R‐squared values as marginal and conditional *R* ^2^ statistics (according to Nakagawa et al., 2017). The marginal *R* ^2^ considers only the variance of the fixed effects, while the conditional *R* ^2^ takes both the fixed and random effects into account. We also provided values of part (semi‐partial) *R* ^2^ as the metrics of variance explained uniquely by a particular predictor.

All statistical analyses were made using packages lmerTest (Kuznetsova et al., 2017), sjPlot (Lüdecke, 2020), and partR2 (Stoffel et al., 2020) in R software.

## 3 RESULTS

Preliminary analyses revealed that Si concentration in sedges, vole density, and groundwater level varied significantly between years of study (*p* < .001 for each of the three variables; Table 1, Figure 1).

**TABLE 1:** Estimates of model parameters with confidence intervals for the effect of water level in spring in year *t*, Si concentration in rhizomes in year *t*, and vole density in year *t* − 1 and year *t*, on the autumn concentration of Si in leaves

Predictors	Estimates	CI	*p*	partR2
(Intercept)	0.75	0.56 to 0.93	<.001	
Vole density in year *t* − 1	0.04	0.00 to 0.09	.056	0.095
Vole density in year *t*	0.03	−0.02 to 0.09	.224	0.030
Si_rhiz_	−0.07	−0.16 to 0.01	.096	0.027
Water level in spring	0.47	0.13 to 0.80	.006	0.133
Marginal *R* ^2^	0.226			
Conditional *R* ^2^	0.287			

**FIGURE 2:**
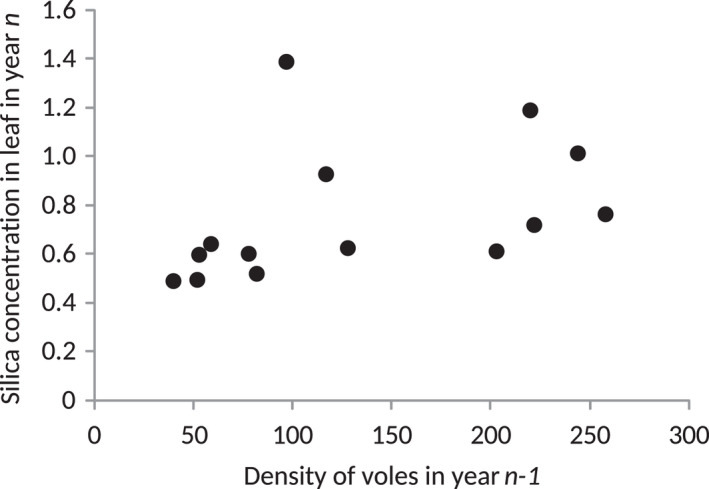
Association between previous year's vole density (*n* − 1) and the current‐year concentration of Si in leaves (Si_leaf_)

GLMM revealed that autumn Si_leaf_ was positively affected by the groundwater level in the spring of the same year (Table 1). The same analysis also revealed the positive effect of the population density of voles recorded in the previous autumn (year *t* − 1; Table 1, Figure 2). Although this effect did not reach statistical significance, it nevertheless explained 9.5% out of 22.6% of Si_leaf_ variation accounted for by all fixed factors (Table 1).

The concentration of Si in rhizomes (Si_rhiz_) was significantly positively affected only by the groundwater level in spring, whereas the effect of vole density in the same year (*t*) and previous year (*t* − 1) was weak and not significant. All fixed effects explained 77.7% of Si_rhiz_ variation (Table 2). When the spring water level was removed from the model, all remaining fixed effects explained only 16.9% of Si_rhiz_ variation and density of voles explained alone 16.1% of Si_rhiz_ variation. In the resulting model, the density of voles in year *t* − 1 became marginally significant (GLMM, coefficient estimate = 0.12, CI: 0.00 – 0.25, *p* = .053).

**TABLE 2:** Estimates of model parameters with confidence intervals for the effect of water level in spring in year *t*, Si concentration in leaves in year *t*, and vole density in year *t* − 1 and year *t*, on the autumn concentration of Si in rhizomes

Predictors	Estimates	CI	*p*	partR^2^
(Intercept)	0.70	0.29 to 1.12	.001	
Vole density in year *t*−1	0.03	−0.06 to 0.13	.484	0.011
Vole density in year *t*	0.06	−0.04 to 0.15	.230	0.029
Si_leaf_	0.01	−0.18 to 0.20	.900	0.000
Water level in spring	1.46	0.83 to 2.10	<.001	0.609
Marginal *R* ^2^	0.777			
Conditional *R* ^2^	0.909			

Neither Si_rhiz_, Si_leaf_, and vole density in year *t* − 1 nor water level in the current year significantly affected the density of the vole population in the current year (*t*) (Table 3).

**TABLE 3:** Estimates of model parameters with confidence intervals for the effect of water level in spring in year *t*, Si concentration in leaves and rhizomes in year *t*, and vole density in year *t* − 1 and year *t*, on the autumn density of voles in year *t*

Predictors	Estimates	CI	*p*	partR2
(Intercept)	−0.40	−0.95 to 0.16	.161	
Vole density in year *t*−1	−0.20	−0.44 to 0.03	.094	0.268
Si_rhiz_	0.10	−0.19 to 0.39	.506	0.059
Si_leaf_	0.06	−0.19 to 0.30	.637	0.001
Water level in spring	−0.05	−0.91 to 0.81	.903	0.000
Marginal *R* ^2^	0.269			
Conditional *R* ^2^	0.792			

## 4 DISCUSSION

Our long‐term study revealed that (1) the amount of Si in leaves was positively related to the current‐year water level in spring and, to a smaller extent, vole population densities from the previous year; (2) spring groundwater level had a strong and positive influence on rhizome Si concentration, but (3) neither Si concentration in leaves or rhizomes nor water level affected the root vole population density. Thus, our findings partly corroborated the results of an earlier study by Wieczorek, Zub, et al. (2015) carried out on the same field study system. This corroboration is important, because the findings presented in Wieczorek, Zub, et al. (2015) have been questioned by Soininen et al. (2017) on statistical grounds.

**FIGURE 3:**
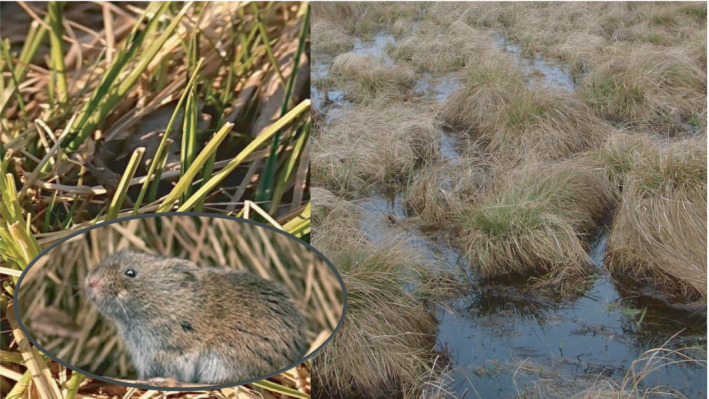
(a) Root vole (*Microtus oeconomus*)—the main herbivorous rodent species living in open areas in Biebrza National Park (BNP), Poland; (b) visible signs of voles grazing on sedge leaves; and (c) typical habitat of root voles in BNP with frequent spring floods in homogenous sedge wetlands

The mechanism of silicification of grasses caused by vole grazing was observed in studies conducted both in a laboratory (Reynolds et al., 2012) and in a landscape‐scale setting (Ruffino et al., 2018). The question therefore arises of why so few confirmations of silica‐induced defense mechanisms in grasses generated by the herbivorous mammals are detected in the wild, while it is so readily detected in laboratory experiments? The most intuitive explanation is that the diet of wild herbivores in natural grasslands is much more diverse than in laboratory studies, which results in insufficient grazing pressure to induce defense mechanisms in a given plant species. Fortunately, in our study system in the Biebrza National Park, homogenous meadows consist almost exclusively of the one Carex species—the tussock sedge—constituting a primary food source of voles (Gębczyńska, 1970). Therefore, this simplest possible one plant–one vertebrate herbivore system is best suited for testing the existence of induction of silicon deposition as a defense mechanism against grazing (Figure 3).

The second possibility is that the elevation in Si concentration in plants is most detectable at high densities of herbivorous mammals, which is easily replicated within laboratory settings (e.g., Massey & Hartley, 2006) but difficult to capture in natural ecosystems. This may explain why the field studies conducted by Soininen et al. (2013) and Quigley et al. (2020) did not reveal consistent relationships between plant silicon concentrations and grazing. The results of our study and other two field experiments (Ruffino et al., 2018; Wieczorek, Zub, et al., 2015) indicate that such relationships are only detectable following response of plants to, especially, high herbivore densities, in this study, above 200 individual/ha, as predicted by the plant defense hypothesis (Haukioja, 1980; Underwood, 1999). This is well illustrated by the changes in water level, Si concentration, and density of voles in the course of our study (Figure 1). After particularly high root vole densities (>200 individuals per hectare) in 2008, there was a notable increase in Si_leaf_ in 2009. Such phenomenon was not observed after 2014 and 2015, years with similarly high vole densities (Figure 1). A putative explanation is that in those years, the water table was low, and therefore, the sedges were unable to accumulate enough Si in response to the high herbivory pressure in the previous years.

Indeed, the key factor affecting the plant–herbivore interaction in our study system is the groundwater level. The uptake and deposition of silicon in wetland plants is driven by hydrological and climatic factors (Schoelynck et al., 2014; Struyf & Conley, 2009; Struyf et al., 2010), because silicic acid uptake in grasses is largely passive and determined by transpiration rate (Sangster et al., 2001, but see Quigley et al., 2020). Wieczorek, Zub, et al. (2015) found that groundwater level positively affects Si_rhiz_. This result has been questioned by Soininen et al. (2017) who asserted that the effect of the groundwater level in Wieczorek, Zub, et al. (2015) study cannot be statistically separated from that of the vole density. Our present analysis, carried out on a much larger data set, allowing for an effective statistical control of the collinearity between independent factors, did support the existence of a strong positive effect of the groundwater level on Si accumulation in leaves and rhizomes (Table 1 and Table 3, respectively). However, we found no evidence that Si_rhiz_ and Si_leaf_, in the same year, are correlated, while Si_rhiz_ in consecutive years was positively correlated. Thus, the dynamics of Si deposition in leaves and rhizomes follow different paths, although in both plant parts, it is driven by the prevailing water regimen (Tables 1 and 2).

Although high grazing pressure of voles elevated Si concentration in sedge's leaves in an apparent delayed density‐dependent manner, it did not affect vole population densities between years. In agreement with this finding, Wieczorek, Zub, et al. (2015) found that the winter survival of voles was not associated with vole fecal Si concentration. In principle, this concentration should be correlated with Si_rhiz_, because the Si_rhiz_ is correlated between subsequent years of study and thus should also faithfully reflect winter Si concentration in rhizomes being the food base of overwintering voles. The lack of the effect of silicification of sedges on the vole population dynamics was likely due to low Si_leaf_ and Si_rhiz_, which in most years of our study remained at the level of less than 1% of dry mass (Figure 1). This level was 3–6 times lower than that reported in leaves of *Deschampsia caespitosa* by Massey et al. (2008)—a study demonstrating negative effect of plant silicification on the population growth and individual performance of voles (*Microtus agrestis*). Likewise, Wieczorek, Szafranska, et al. (2015) study demonstrating the abrasive effect of silica on intestinal villi of voles used sedge‐based diet containing 1.87% of Si in dry mass—a concentration higher than that reported here.

## 5 CONCLUSIONS

Our results revealed that the plant–herbivore–water–regime nexus is more complex than has been described from laboratory (Seldal et al., 1994) and enclosure experiments (Agrell et al., 1995). We demonstrated that silicification process of rhizomes of sedges in our study area is mainly driven by the hydrological cycles. Si concentration in leaves appears to be dependent on the groundwater level, and slightly positively affected by the previous year's vole population density. However, this effect does not create a feedback loop predicted by the plant defense hypothesis, as silicification of sedges was insufficient to negatively affect the root vole population dynamics. Therefore, future research carried out on the plant defense hypothesis should aim at disentangling whether anti‐herbivore protection is dependent on threshold values of the herbivory pressure. Above all, however, studies on Si accumulation must take into account the effect of water‐mediated Si availability.

## CONFLICT OF INTEREST

The authors declare no conflict of interest.

## AUTHOR CONTRIBUTIONS

**Zbigniew Borowski:** Conceptualization (equal); Data curation (equal); Funding acquisition (lead); Investigation (equal); Methodology (equal); Project administration (lead); Writing‐original draft (equal); Writing‐review & editing (equal). **Karol Zub:** Conceptualization (equal); Data curation (equal); Formal analysis (lead); Investigation (equal); Methodology (equal); Project administration (equal); Supervision (equal); Validation (equal); Visualization (lead); Writing‐original draft (equal); Writing‐review & editing (equal). **Marcin Sulwiński:** Formal analysis (equal); Methodology (equal). **Małgorzata Suska‐Malawska:** Resources (supporting). **Marek Konarzewski:** Conceptualization (equal); Formal analysis (equal); Investigation (equal); Methodology (equal); Project administration (equal); Supervision (equal); Validation (equal); Visualization (equal); Writing‐original draft (equal); Writing‐review & editing (equal).

## Data Availability

Data used for analyses are available on Dryad Digital Repository at https://doi.org/10.5061/dryad.cz8w9gj4h.
